# Multi-Objective Optimization Design of Ladle Refractory Lining Based on Genetic Algorithm

**DOI:** 10.3389/fbioe.2022.900655

**Published:** 2022-06-15

**Authors:** Ying Sun, Peng Huang, Yongcheng Cao, Guozhang Jiang, Zhongping Yuan, Dongxu Bai, Xin Liu

**Affiliations:** ^1^ Key Laboratory of Metallurgical Equipment and Control Technology of Ministry of Education, Wuhan University of Science and Technology, Wuhan, China; ^2^ Research Center for Biomimetic Robot and Intelligent Measurement and Control, Wuhan University of Science and Technology, Wuhan, China; ^3^ Hubei Key Laboratory of Mechanical Transmission and Manufacturing Engineering, Wuhan University of Science and Technology, Wuhan, China; ^4^ Hubei Jingmen Wusan Machinery Equipment Manufacturing Co., Ltd, Jingshan, China

**Keywords:** genetic algorithm, multi-objective optimization, thermal insulation performance, ladle refractory lining, service life

## Abstract

Genetic algorithm is widely used in multi-objective mechanical structure optimization. In this paper, a genetic algorithm-based optimization method for ladle refractory lining structure is proposed. First, the parametric finite element model of the new ladle refractory lining is established by using ANSYS Workbench software. The refractory lining is mainly composed of insulating layer, permanent layer and working layer. Secondly, a mathematical model for multi-objective optimization is established to reveal the functional relationship between the maximum equivalent force on the ladle lining, the maximum temperature on the ladle shell, the total mass of the ladle and the structural parameters of the ladle refractory lining. Genetic algorithm translates the optimization process of ladle refractory lining into natural evolution and selection. The optimization results show that, compared with the unoptimized ladle refractory lining structure (insulation layer thickness of 0 mm, permanent layer thickness of 81 mm, and working layer thickness of 152 mm), the refractory lining with insulation layer thickness of 8.02 mm, permanent layer thickness of 76.20 mm, and working layer thickness of 148.61 mm has the best thermal insulation performance and longer service life within the variation of ladle refractory lining structure parameters. Finally, the results of the optimization are verified and analyzed in this paper. The study found that by optimizing the design of the ladle refractory lining, the maximum equivalent force on the ladle lining, the maximum temperature on the ladle shell and the ladle mass were reduced. The thermal insulation performance and the lightweight performance of the ladle are improved, which is very important for improving the service life of the ladle.

## 1 Introduction

Refractory lining is an important part of the ladle, mainly including working layer, permanent layer and insulation layer, and its thickness affects the ladle shell temperature and ladle lining force. The structural composition of the refractory liner affects the ladle lightweight performance and insulation performance. The greater the thickness of the working layer, the lower the temperature of the ladle shell, but at the same time it will increase the weight and production cost of the ladle. In order to ensure that the ladle shell temperature and lining stress are within the permitted range, the thickness of the working layer cannot be too large or too small, and a balance point needs to be found between the two. At present, some scholars have reported on the optimal design of the ladle in order to improve the service life of the ladle, but they mainly focus on the use of the lining material, which is not only cumbersome, but also costly. There are few introductions about improving the service life of the ladle by optimizing the lining structure. The emergence of the bionic intelligent algorithm provides a new idea for the optimization of the refractory lining structure, which transforms the solution process into an optimization problem of finding the optimal solution, which not only simplifies the solution process, but also improves the solution efficiency. Multi-objective genetic algorithm (MOGA) has the advantages of high accuracy, fast solution speed and stronger applicability in multi-objective problem solving and parameter optimization. Many groups of Pareto optimal solutions are obtained by multi-objective optimization, but not all Pareto optimal solutions meet the actual design requirements. Therefore, it is necessary to find the optimal solution from these optimal solutions. Biological evolution is a very magical process. It produces excellent species through the laws of nature such as selection, elimination, and mutation. Genetic Algorithm (GA) is a computer abstracted from the selection and evolution process of nature. This paper is based on ANSYS finite element analysis software to optimize the design of ladle refractory lining, which is an organic combination of mechanical design and computer simulation technology (Dhaduti and sarganachari, 2020; Mysore and patil, 2021). The parametric solid modeling of the ladle is performed using ANSYS Workbench software, and the model is imported using the seamless connection between the software (Nimbagal and Banapurmath, 2021; Patil and Bano, 2021). The ANSYS software allows direct input of structural geometry and finite element meshing, as well as direct input of constraints and load data, which makes the tedious task of filling in data files directly intuitive and easy, and makes it easy to detect errors in the input process and correct them in time. It can be applied to various fields of structural analysis and coupling between fields (Patil and Banapurmath, 2019; Patil and Banapurmath, 2020).

Aiming at the optimization problem of ladle refractory lining structure, this paper proposes an optimization design method based on multi-objective genetic algorithm, which has the following innovations:

1) In this paper, a multi-objective genetic algorithm is introduced to solve the optimization problem of the ladle refractory lining structure, and the genetic algorithm transforms the optimization process of the ladle refractory lining into natural evolution and selection. The effects of different working layer thicknesses, permanent layer thicknesses and insulating layer thicknesses on the temperature field of the ladle shell and the stress field of the ladle lining are analyzed, and find the optimal solution for the thickness of the working layer, the permanent layer thickness and the insulating layer thickness. This method of finding the optimal solution is faster and more accurate.

2) In this paper, the response surface method and genetic algorithm are combined to optimize the structural dimensions of the ladle lining. The method retains the characteristics of the theoretical calculation method, constructs the parameters of the thickness of the working layer, the thickness of the permanent layer and the thickness of the insulating layer, and outputs the response surface function of the natural frequency. The response surface function is used to replace the original finite element model, and then the implicit relationship between the size parameters of the lining components and the output natural frequency is converted into a simple fitting polynomial, so that the optimization algorithm avoids the need to find the optimal solution. The repeated iterative calculation of the entire model saves calculation time while ensuring the solution accuracy.

The remainder of this article is described below. Section 2 introduces the current research status of multi-objective optimization at home and abroad, the research status of the lightweight performance of the ladle and the research status of the thermal insulation performance of the ladle. Section 3 provides a detailed description of the process of finite element model establishment, selection of material parameters, setting of boundary conditions and thermal stress analysis of the ladle. Section 4 introduces the process of ladle lining optimization design, the selection of response surface experimental design method and the establishment of response surface model. Section 5 uses the multi-objective genetic algorithm to optimize the response surface model, obtains the optimization results, and conducts verification analysis. Compared with the data before optimization, it can be seen that this method achieves the optimization effect. Finally, the conclusion part of this paper is introduced.

## 2 Related Work

Multi-objective problems have been studied for decades. Since the late 1980s, the research on multi-objective genetic algorithm MOGA has entered a flourishing period in academia (Cheng et al., 2021; Hao and Wang, 2022). In the past 10 years, papers on multi-objective genetic algorithms have emerged one after another (Cheng et al., 2020; Sun and Yang, 2021). The reason why MOGA research has such a good momentum is mainly because of its broad application prospects in engineering fields (Shi and Huang, 2022b; Wu and Jiang, 2022). On the one hand, the theory of genetic algorithm used to solve multi-objective problems has become more and more mature (Luo and sun, 2020; Liu and Xu, 2022a); on the other hand, with the rapid development of science and technology, many practical problems are the joint optimization problems of multiple objectives, and there are contradictions between the sub-objectives relationship (Li and Li, 2020; Liu et al., 2022b), the problem is more high-dimensional and complex (Tao and Liu, 2022a; Liu and Li, 2022c). The parallelism of the genetic algorithm in solving, the superior global optimization of the algorithm and the fact that the algorithm itself is not limited by the continuity of the function determine that it can be well used in solving high-dimensional complex problems (Alqaili and Qais, 2021; Panagant and pholdee, 2021).

Multi-objective optimization is when multiple objectives need to be achieved in a single scenario (Szlapczynska and szlapczynski, 2019), and optimizing one objective is often at the expense of degrading the others due to the inherent conflict that easily exists between the objectives (Sun and Hu, 2020a; Tong Ma, 2022). Therefore, it is difficult to get the unique optimal solution (Sun and hu, 2020b; Tian and cheng, 2020). Instead, it is necessary to coordinate and compromise among them to make the overall goal as optimal as possible. In practical engineering optimization problems, many design problems do not have only one design index requirement (Li and Li 2016; Bai et al., 2022; Liu et al., 2022d). For example, when designing a new type of ladle, it is usually hoped that the ladle has light weight, corrosion resistance, good thermal insulation performance, and long service life (Tan and sun, 2020; Tao and Wang, 2022b; Wang and Huang, 2022). This kind of problem that requires multiple design variables to achieve optimality is a multi-objective optimization problem (Santos and Moreira, 2018; Srivastava and chattopathyay, 2021). In recent years, the multi-objective optimization problem has been paid more and more attention by scholars at home and abroad. The application of multi-objective genetic algorithm in the optimization design of some mechanical fields is also increasing (Chen and Yin, 2021a; Chen and jin, 2022a). Many complex optimization problems in engineering can be solved by multi-objective optimization theory (Sun and weng, 2020c).

MOGA is widely used in various fields: scientific field, economic field, engineering field, etc (Jiang et al., 2019a; Sun and tian, 2022a). And has also made good progress, such as robot path planning, optimal control (Ma and Zhang, 2020; Qi and Jiang, 2020), mechanical structure case optimization design, mobile network planning, humanoid robot hub Design of neural motion controller, optimal design of solid rocket motor (Jiang et al., 2019b; Sun and Zhao, 2022b), optimal design of postman path, optimal design of multi-sensor multi-object tracking data association problem, flexible multi-objective scheduling problem (Chen and peng, 2021b; Chen and Rong, 2022b), vehicle scheduling, etc.

The density-based multi-objective evolutionary algorithm is proposed by researchers. The main idea of the algorithm is to quantify the degree of mutual influence between any two individuals in the population (Chang et al., 2018; Jiang and Li, 2021a), so as to define the degree of aggregation between individuals, and to maintain the diversity of the population (Jiang kong, 2006; Jiang and Li, 2021b). The simulation test of the numerical example verifies the effectiveness of the method (Liao and Li, 2020; Liu and jiang, 2021; Najm and EL-Hassan, 2021). In view of the fact that the Pareto optimal solution obtained by multi-objective genetic algorithm in solving some complex high-dimensional multi-objective optimization problems is easily affected by the disturbance of design parameters (Yu and Li, 2020; Yang and Jiang, 2021; Yun and sun, 2022), the concept of robustness and the proposal of an improved robust multi-objective optimization method are used by some scholars proposed (Li and Kong, 2012; Li and Liu, 2015a; Jiang et al., 2019c). On the basis of the classical fitness function expectation and variance, the expectation and variance are effectively combined, and the particle swarm algorithm is integrated at the same time (Yu and Li, 2019; Sun and xu, 2020d). The simulation case results verify that the Pareto optimal solution obtained by this method is more robust (Chen and Qiu., 2022c).

In the field of ladle, due to the rapid development of traditional manufacturing, the role of ladle is no longer just a container for transporting and casting molten steel (Li and Liu, 2015b; Li and Jiang, 2019), it also plays a role in thermal insulation of molten steel and guarantees for subsequent operations (Boikov et al., 2019; Bilen, 2020). Due to the many steps of steelmaking and the overload (Mazumdar and Guthrie, 2020; Kadam and brooks, 2021), the molten steel needs to stay in the ladle for a longer time, and the operation of the ladle is increased during the turning process when the molten steel is attacked (Liu and Jiang, 2022f; Li and Kong, 2006; Li et al., 2017). The molten steel time and casting time in the ladle increase exponentially, and a better lining refractory and thermal insulation lining structure is needed to withstand the erosion of high-temperature molten steel. Insulating refractory materials are used to achieve the super thermal insulation performance of the ladle (Li et al., 2015; Li and Qu, 2013). The ladle cover is also used by some scholars to analyze the influence on the thermal insulation performance of the ladle (Zhang and zho, 2020; Liu and Jiang, 2021), and it is found that the addition of the cover can effectively improve the thermal insulation performance of the ladle (Chakraborty and sinha, 2019; Choi and kim, 2019), and can effectively increase the temperature of the molten steel during tapping. Although the thermal insulation of the ladle has been analyzed and studied in detail by the above-mentioned researchers (Sun and tian, 2020a; Weng and sun, 2021), the longevity and lightweight performance of the ladle have not been considered. Some scholars even sacrifice these two aspects to achieve the effect of thermal insulation (Xiao and Li, 2021; Yan and conejo, 2022). In line with the requirements of contemporary development, it will also bring hidden dangers to steelmaking and cause safety accidents (Zhu, 2013; Zhang and Nie, 2017).

The optimal design of the ladle has been reported by many scholars, but it mainly focuses on the use of lining materials (Zeng and Wang, 2022; Zhang and Wang, 2022). After parametric modeling of the ladle, the response surface method in finite element optimization design is used to optimize the size of the working layer, permanent layer and insulating layer of the refractory lining based on MOGA, thereby improving the use of the ladle life. This research work is of great significance.

## 3 Finite Element Analysis of Steel Ladle

### 3.1 Structure and Physical Parameters of Steel Ladle

**FIGURE 1:**
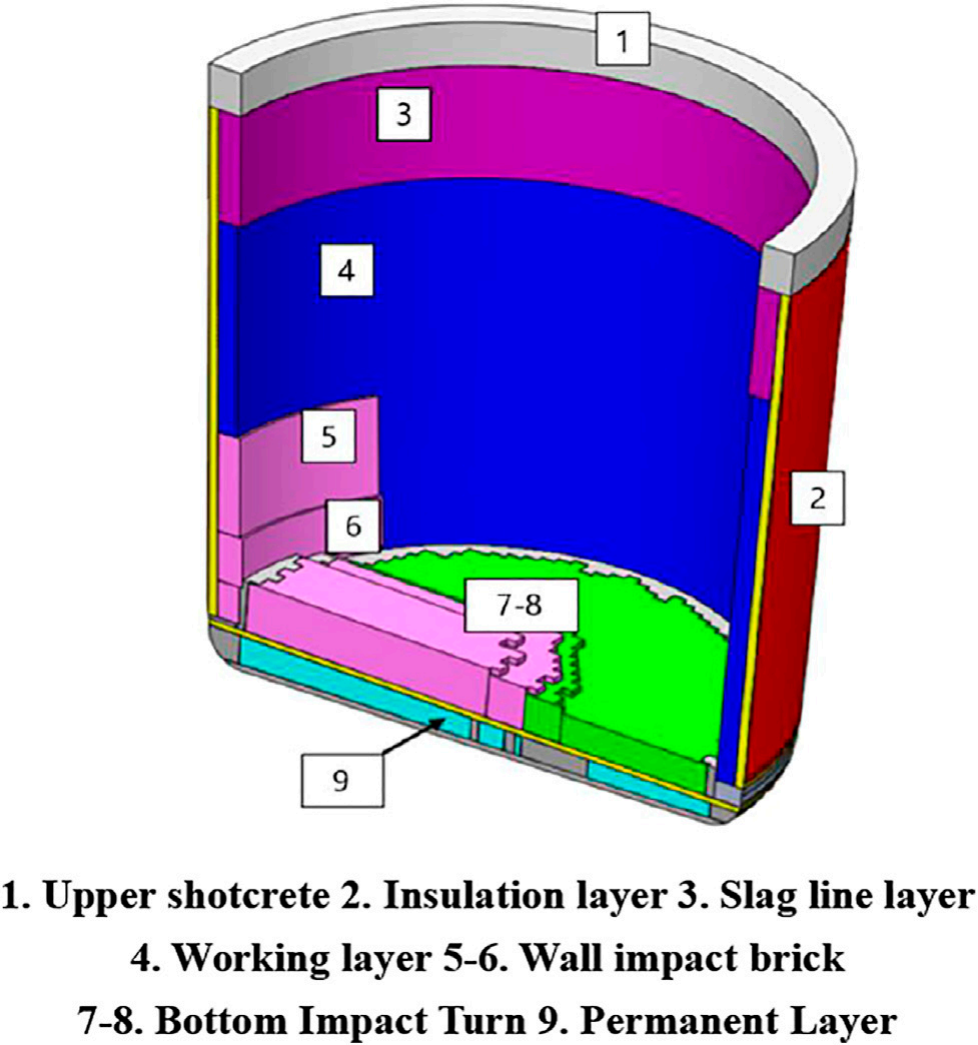
Ladle lining structure composition.

The composition of the ladle lining structure is shown in Figure 1 (Li and kong, 2012). The main function of the upper part of the shotcrete is to strengthen the ladle and play a supporting role. The wall impact brick and bottom impact brick are set to reduce the damage of steel to the ladle bottom and ladle side walls during transportation. In order to improve the speed and ensure the accuracy of optimization of ladle lining, this paper simplifies the ladle lining by ignoring the upper shotcrete, wall impact brick and bottom impact brick.

**FIGURE 2:**
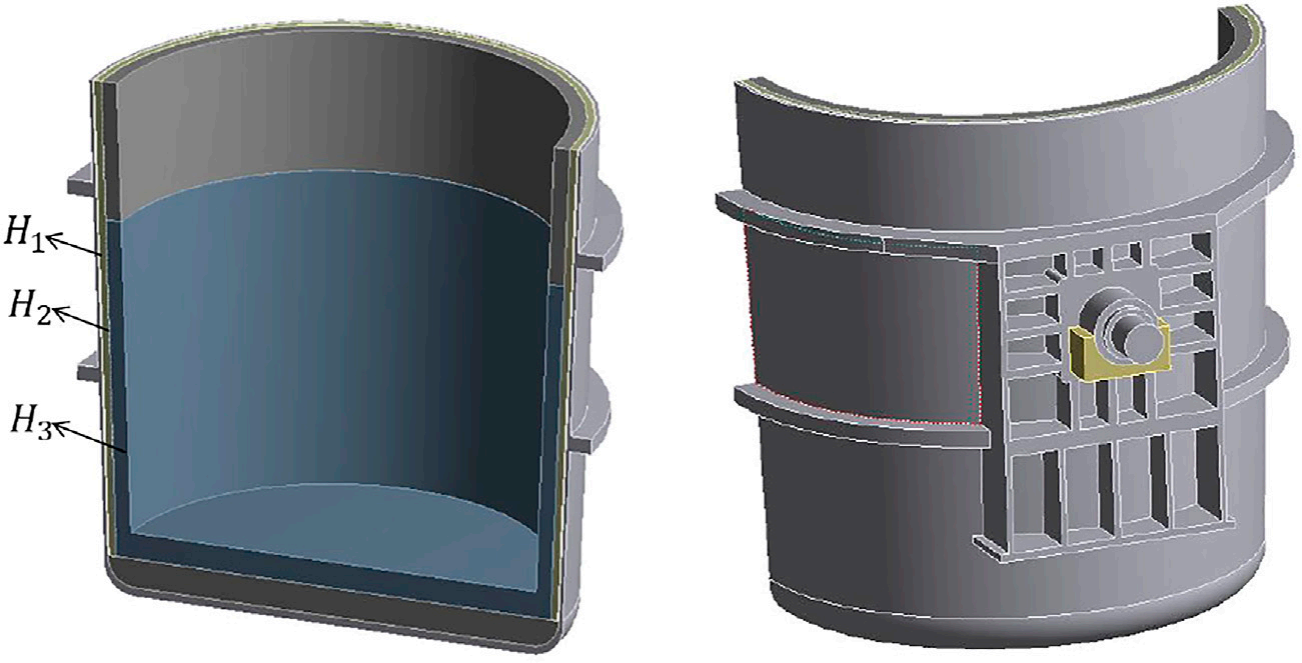
New parametric model of steel ladle.

In the optimization analysis of each structure of the refractory lining of the new ladle, this paper uses ANSYS Workbench software to establish a parametric model of the new ladle, as shown in Figure 2, which is the finite element analysis software version 2020 R2. In the figure, H_1_, H_2_ and H_3_ are the design variables to be optimized, H_1_ is the thickness of the insulation layer, H_2_ is the thickness of the permanent layer and H_3_ is the thickness of the working layer. The ladle height is 4917 mm, ladle shell thickness is 40 mm, ladle width is 2409 mm, waistband trunnion upper waistband thickness is 150 mm, upper waistband lower edge is 3780 mm from the bottom of the ladle, waistband trunnion lower waistband thickness is 150 mm, lower waistband lower edge is 2005 mm from the bottom of the ladle, ladle initial mass is 58192 kg, and volume is 14.35 m^3^. Supplementary Table S1 shows the variation range of the dimensions of the three design variables.

The working layer is the temperature load bearing object, the ladle shell is in contact with the surrounding air, the permanent layer is located between the working layer and the insulating layer, and the insulating layer is located between the permanent layer and the ladle shell. Due to the high lining temperature of the working layer and the low temperature of the ladle shell, the heat will be transferred from the working layer to the lining of the permanent layer and the ladle shell, and the heat transfer mode is conduction. The ladle shell dissipates heat to the surrounding air by convection and radiation. The main purpose of increasing the permanent layer and insulating layer is to reduce the temperature of the steel cladding and prevent the high-temperature creep deformation of the steel cladding. With the prolongation of use time, cracks and damage will occur, reducing the service life of the ladle.

When performing numerical simulations of temperature and stress fields in ANSYS, it is necessary to specify the physical parameters of the material and the boundary conditions of the model, which are crucial for the accuracy of the calculation results. Physical parameters of materials such as thermal conductivity and specific heat are temperature dependent and their values change with temperature. However, since the experimental data of thermal conductivity of materials at high temperature are not easy to obtain. Therefore, it is treated as a constant in this thesis, and the thermal conductivity is considered as a constant independent of temperature. In the thermal stress analysis, the physical parameters involved mainly include material density, thermal conductivity, specific heat, elastic modulus, and Poisson’s ratio, as shown in Table 1 (Li and Qu, 2013; Li and Liu, 2015c). The ladle shell material is selected as 15CrMoR (Zhang and zhong, 2019).

**TABLE 1:** The main physical parameters of the ladle.

Refractory	Coefficient of Expansion $\left(\text{α}\times 10^{-6}K^{-1}\right)$	Elastic Modulus (MPa)	Poisson’s Ratio	Density kg/mm^−3^	Specific heat (J/kg k)	Thermal conductivity (w/m ⋅ k)
Working layer (aluminum magnesium carbon)	8.5	6300	0.21	2.95e-6	1200	1.6
Permanent layer (high aluminum)	5.8	5700	0.21	2.8e-6	1320	0.9
Slag line layer (magnesium carbon)	15	8000	0.3	2.9e-6	1080	1.55
Ladle shell	13	206000	0.3	7.85e-6	600	31
Insulation layer	3	2000	0.01	0.26e-6	919	0.122

### 3.2 Cell Selection and Mesh Division

**FIGURE 3:**
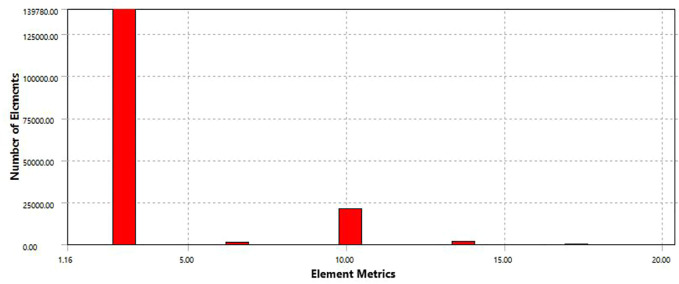
Mesh metric aspect ratio.

During the temperature field analysis of the ladle, the mesh division takes symmetry to improve the computational efficiency because the simplified ladle model is symmetrical. Set the contact type as friction contact and the friction coefficient as 0.2. The selected cell type is Quad4 Node55 cell in Thermal Solid, and the cells selected for the contact area are CONTA175 cell and TARGE169 cell. When dividing the mesh, the control cell size is 0.1m, mainly tetrahedral. Because tetrahedral meshes adapt well to complex geometries, they are mostly used for free meshing and can generate meshes quickly. A total of 163,094 units and 263,305 nodes are divided, and the mesh division model is shown in Supplementary Figure S1. As can be seen from Figure 3, the grid cell aspect ratio is above 0.3, basically in the range of 1.16–5. The grid cell quality is high and meets the requirements of solution accuracy.

### 3.3 Thermal Analysis of Ladle

Different forms of heat transfer occur during drying, preheating and casting of the ladle. According to the heat transfer mechanism distinction, it can be divided into heat conduction, heat convection and heat radiation. Heat conduction mainly occurs between the refractory lining of the ladle and between the lining and the ladle shell. Convection heat transfer as well as radiation heat transfer occurs between the ladle shell and air, and between the refractory liner and the steel (Li and Qiu, 2013).

1) Heat conduction

Inside the same object or between different objects there is a temperature difference, heat transfer along the object from the high temperature part to the low temperature part of the way called heat transfer, the essence is the thermal movement of microscopic particles (Li and Jiang, 2019). The formula for heat conduction is shown in Eq. 1.

Φ=λAΔT/δ (1)

Where: Φ is the heat flow through a uniform plane of the object, W ; ΔΤ is the temperature difference between the two sides of the object, Cο ; λ is the thermal conductivity, W/(m·K) ; δ is the thickness of the plate, m ; Α is the area of the plate, $m^{2}$ .

2) Thermal convection

Thermal convection is the phenomenon of heat transfer in space using the hot particles of the flowing medium. Convective heat transfer often occurs between fluid and fluid, fluid and solid surface (Li et al., 2019). The equation of thermal convection is shown in Eq. 2.

Φ=ΑαΔΤ (2)

Where: Φ is the heat flow through a uniform plane of the object, W ; ΔΤ is the temperature difference between the two sides of the object, K ; Α is the surface area of convective heat transfer, $m^{2}$ ; α is the surface convective heat transfer coefficient, $W/\left(m^{2}\cdot K\right)$ .

3) Thermal radiation

Heat radiation is in the form of electromagnetic waves to achieve the transfer of heat, does not require a medium, belongs to the non-contact heat transfer. Heat radiation exists between the ladle components and between the ladle and air. When the ladle is subjected to transient heat transfer analysis, the temperature field T (x, y, z) satisfies the differential equation in the right angle coordinate system as shown in Eq. 3 (Zhang and Zhu, 2020):

$$\rho c\frac{\partial T}{\partial t}=\frac{\partial }{\partial x}\left(K_{x}\frac{\partial T}{\partial x}\right)+\frac{\partial }{\partial y}\left(K_{y}\frac{\partial T}{\partial y}\right)+\frac{\partial }{\partial z}\left(K_{z}\frac{\partial T}{\partial z}\right)$$ (3)

In the formula: ρ is the density of the material, $kg/m^{2}$ ; c is the specific heat of the material, J/(kg⋅K) ; t is the time, s ; $K_{x}$ , $K_{y}$ , and $K_{z}$ are the thermal conductivity of the material in different directions, W/(m⋅K) respectively.

4) Determination of the boundary conditions.

In the finite element analysis of the ladle, the temperature and stress changes of the ladle shell and refractory liner during the casting process are mainly analyzed. Considering the complex changes of the ladle thermal state and its influencing factors, the following assumptions are made: 1) Adding trunnion auxiliary blocks to simulate the ladle spreader or support member. During the casting process, elastic constraints in the *X*, *Y* and *Z* directions are applied to the nodes in the plane where the trunnion is in contact with the auxiliary block; 2) Ignoring the contact thermal resistance between different refractory materials; 3) Not considering the stratification of the steel temperature in the ladle, the steel temperature is uniformly distributed, and the steel temperature change is neglected in the pouring stage, and the steel temperature is 1,600°C.

During the casting cycle of the ladle, there are only two ways to dissipate heat from the outer surface of the ladle shell. One is the thermal convection between the ladle shell and the surrounding air, and the other is the thermal radiation between the ladle shell and the surrounding environment. Ambient temperature is 20°C. That is, type 3 boundary conditions are used (Li and Liu, 2015a).

Generally, the natural convection coefficient between the air and the steel cladding is 5–10 $W/\left(m^{2}gK\right)$ (Li and Liu, 2015b), and the more accurate calculation is

$$h=1.826{\left[\frac{T_{s}}{T_{s}-T_{a}}\right]}^{1/3}$$ (4)

Among them, h is the convective heat transfer coefficient, $T_{s}$ is the temperature of the ladle shell, and $T_{a}$ is the temperature of the surrounding air.

The ability of an object to radiate heat depends primarily on the temperature of the object (Li and Liu, 2015c; Li et al., 2013). However, due to the highly nonlinear calculation of radiation heat transfer, it takes a lot of calculation time, and a simplified form can be used, that is, the radiative heat transfer is converted into the form of convective heat transfer, which can be replaced by the equivalent convective heat transfer coefficient through the data. Radiant heat transfer. The equivalent convective heat transfer coefficient when the radiative heat transfer between the ladle shell and the surrounding is converted into convective heat transfer can be expressed by formula (5).

$$h_{r}=\varepsilon B\left(T_{\text{s}}^{2}-T_{\text{a}}^{2}\right)\left(T_{s}-T_{\text{a}}\right)$$ (5)

where $h_{r}$ is the equivalent convective heat transfer coefficient, $T_{s}$ is the temperature of the ladle shell, $T_{a}$ is the temperature of the surrounding air, B is the Boltzmann constant, and ε is the emissivity.

The radiation coefficient ε is 0.8 and Boltzmann constant B = 5.67e^−8^. The calculated combined convective heat transfer coefficient is shown in Table 2.

**TABLE 2:** Integrated convective heat transfer coefficient on the outer surface of the ladle.

Temperature of Ladle Shell/°C	20	100	200	300	400	500
Heat transfer coefficient/ $w\cdot m^{-2}\cdot K^{-1}$	14.6	18.8	25.7	36.2	56.6	75

### 3.4 Stress Analysis of Ladle

Various parts of the ladle generate temperature stress in the ladle due to the difference in temperature, and expansion pressure is generated between the various parts of the ladle due to different thermal expansion coefficients and constraints. In the stress field calculation for the ladle, the sequential coupling method is used. The temperature field of the model is calculated first, and then the stress field of the ladle is calculated by applying the temperature field results to the model as the load for the stress field calculation (Li et al., 2015b; Li et al., 2019).

In calculating the ladle thermal stress, the deformation displacement of each point within the ladle is first calculated based on the overall temperature distribution of the ladle and the coefficient of thermal expansion of different parts. Then the strain at each point within the ladle is calculated using the geometric equation (strain-displacement relationship). Finally, the stress at each point in the ladle is calculated according to the physical equation of the ladle material (the relationship between stress and strain) (Li et al., 2015c; Li et al., 2013).

1) The ladle stress field geometric equation-strain-displacement relationship is shown in Eq. 6.

$$\varepsilon =\left[\begin{array}{ccc} \frac{\partial }{\partial x} & 0 & 0\\ 0 & \frac{\partial }{\partial y} & 0\\ 0 & 0 & \frac{\partial }{\partial z}\\ \frac{\partial }{\partial y} & \frac{\partial }{\partial x} & 0\\ 0 & \frac{\partial }{\partial z} & \frac{\partial }{\partial y}\\ \frac{\partial }{\partial z} & 0 & \frac{\partial }{\partial x} \end{array}\right]\upsilon$$ (6)

Where $\varepsilon ={\left[\varepsilon_{\text{x }}\varepsilon_{\text{y }}\varepsilon_{\text{z }}\gamma_{\text{xy }}\gamma_{\text{xz }}\gamma_{\text{yz}}\right]}^{\text{T}}$ is the strain at any point in the ladle. $\upsilon ={\left[\text{u v w}\right]}^{\text{T}}$ , u、v 、w denotes the displacements along *x*, *y* and *z* directions, respectively, m.

2) The physical equation of the ladle stress field - the strain-stress relationship is shown in Eq. 7.

$$\sigma =\frac{E\left(1-\upsilon \right)}{\left(1+\upsilon \right)\left(1+2\upsilon \right)}\left[\begin{array}{cccccc} 1 & \frac{\upsilon }{1-\upsilon } & \frac{\upsilon }{1-\upsilon } & 0 & 0 & 0\\ \frac{\upsilon }{1-\upsilon } & 1 & \frac{\upsilon }{1-\upsilon } & 0 & 0 & 0\\ \frac{\upsilon }{1-\upsilon } & \frac{\upsilon }{1-\upsilon } & 1 & 0 & 0 & 0\\ 0 & 0 & 0 & \frac{1-2\upsilon }{2\left(1-\upsilon \right)} & 0 & 0\\ 0 & 0 & 0 & 0 & \frac{1-2\upsilon }{2\left(1-\upsilon \right)} & 0\\ 0 & 0 & 0 & 0 & 0 & \frac{1-2\upsilon }{2\left(1-\upsilon \right)} \end{array}\right]\varepsilon$$ (7)

Where: E is the modulus of elasticity, MPa; υ is the Poisson’s ratio; ε is the strain.

3) Ladle equilibrium equation.

For the three-dimensional problem, the equilibrium equation of any point in the ladle along the coordinates *x*, *y*, *z* direction is as in Eq. 8.

$$\{\begin{array}{c} \frac{\partial \sigma_{x}}{\partial x}+\frac{\partial \tau_{yx}}{\partial y}+\frac{\partial \tau_{zx}}{\partial z}+f_{x}=0\\ \frac{\partial \tau_{xy}}{\partial x}+\frac{\partial \sigma_{y}}{\partial y}+\frac{\partial \tau_{zy}}{\partial z}+f_{y}=0\\ \frac{\partial \tau_{xz}}{\partial x}+\frac{\partial \tau_{yz}}{\partial y}+\frac{\partial \sigma_{z}}{\partial z}+f_{z}=0 \end{array}$$ (8)

Where $f_{x}$ 、 $f_{y}$ 、 $f_{z}$ is the component of volume force per unit volume of the ladle in *x*, *y* and *z* directions.

## 4 Response Surface Experiment Design and Analysis

### 4.1 Optimization Design Process of Ladle Lining

**FIGURE 4:**
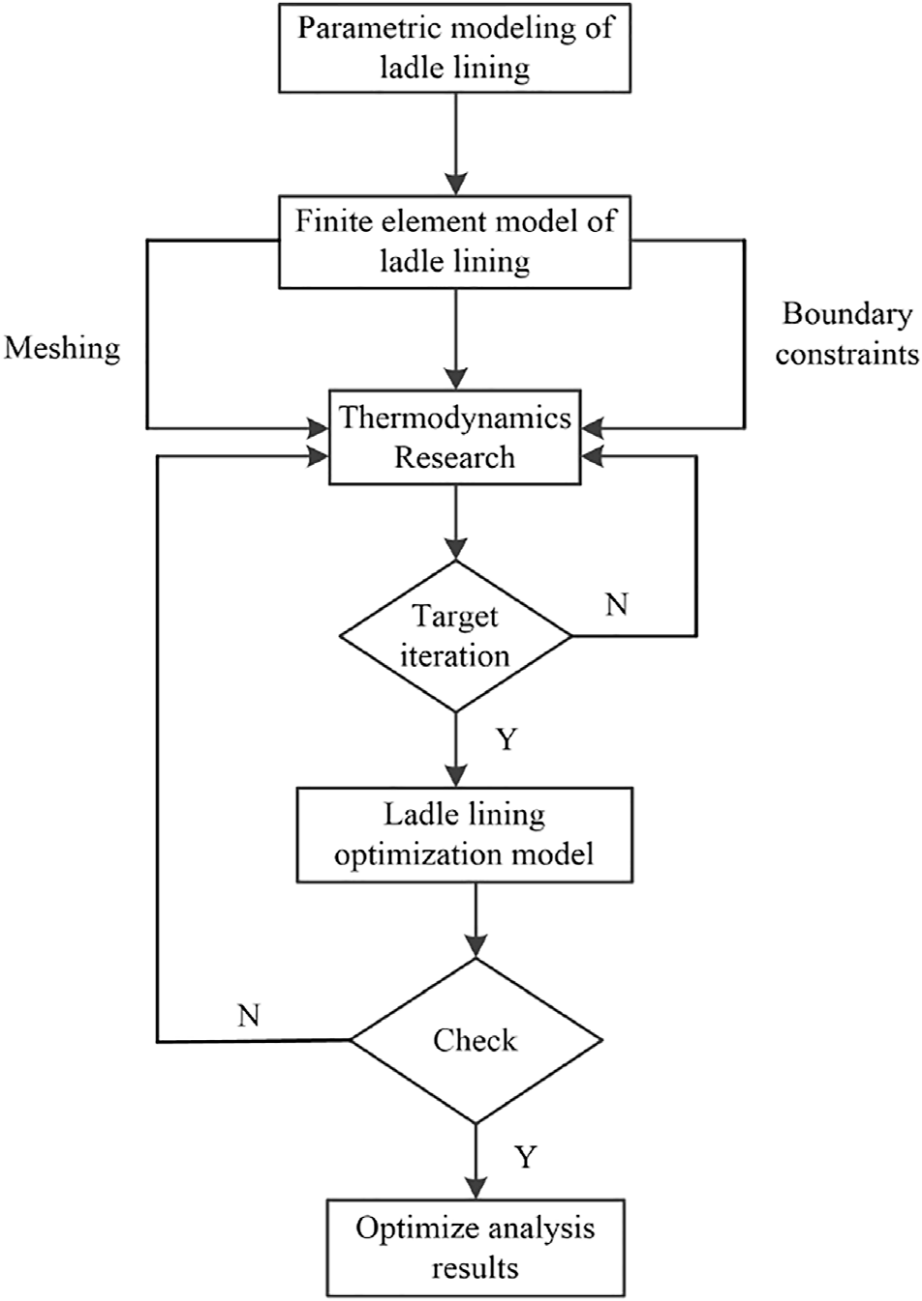
Flow chart of ladle lining optimization.

According to the finite element analysis theory, parametric modeling of the ladle lining is carried out, and ANSYS finite element analysis software is selected to carry out relevant analysis. The lining optimization flow chart is shown in Figure 4.

### 4.2 Choice of Experimental Design Method

On the premise of ensuring that the strength of the ladle lining is satisfied, the lightweight and thermal insulation of the ladle should be ensured as much as possible. Select the maximum equivalent stress of the ladle lining, the maximum temperature of the ladle shell, and the total mass of the ladle as the output parameters, that is, the objective function. The thickness of the insulating layer, the thickness of the permanent layer and the thickness of the working layer are selected as input parameters to be constrained, that is, the design variables. A preferred solution for an optimized design is obtained. When building a response surface model, we first need to conduct experimental design. Common experimental design methods include central composite design (CCD), optimally filled space design (OSFD), Latin hypercube sampling (LHS) and so on. In this paper, OSFD is used to select experimental points. This method is optimized by LHS, and OSFD can evenly distribute design parameters throughout the design space to gain maximum insight into design points with minimum number, compared to CCD focusing on parameters near the design area. It is suitable for complex modeling such as Kriging, nonparametric regression, neural network, etc. The design type selects maximum entropy, which can maximize the covariance of sampling points and reduce the uncertainty of unobservable locations. The sample type is a full quadratic model sample. After the above method is set, a set of experimental points will be automatically generated inside the analysis system, and the iterative solution of this set of experimental points will be performed. The values of the experimental points are shown in Table 3.

**TABLE 3:** Values of experimental points.

Serial Number	Insulation Layer Thickness (mm)	Permanent Layer Thickness (mm)	Working Layer Thickness (mm)	Ladle Quality (kg)	Maximum Temperature of Steel Cladding (°C)	Maximum Stress of lining(MPa)
1	0.5	73.6	159.5	59,057	207.65	43.787
2	7.5	70.4	162.5	59,015	157.30	44.775
3	1.5	81.6	150.5	58,936	198.88	42.426
4	2.5	76.8	138.5	57,575	200.39	43.456
5	4.5	80.0	165.5	60,014	168.89	44.006
6	9.5	78.4	156.5	59,132	145.75	43.319
7	6.5	83.2	144.5	58,542	160.99	43.168
8	8.5	72.0	141.5	57,417	156.34	45.205
9	3.5	68.8	147.5	57,686	196.88	44.341
10	5.5	75.2	153.5	58,663	168.56	43.103

### 4.3 Establishment of Response Surface Model

#### 4.3.1 Selection of Response Surface

Commonly used response surface fitting methods generally include second-order polynomial, kriging, non-parametric regression, neural network and other methods. In this paper, non-parametric regression (non-parametric regression) method is used to establish the response surface. This method has high fitting efficiency and is suitable for fitting large-scale and highly nonlinear sample data (Tao and Huang, 2021; Wei and Wang, 2021; Zhao and Jiang, 2022).

First input sample $X=\left\{x_{1},x_{2},x_{3},...,x_{i}\right\}$ , $x_{i}$ represents the input column vector of N dimension, determine the form of the equation:

Y≤W,X>+c (9)

W in the above formula represents the weight vector, in the general non-parametric case, formula (9) can be expressed as:

$$Y=\sum_{i=1}^{N}(A_{i}-A_{i}^{*})K({\vec{X}}_{i},\vec{X})+c$$ (10)

Where $K\left({\vec{X}}_{i},\vec{X}\right)$ is the kernel map and $A_{i}、A_{i}^{*}$ is the Lagrange multiplier.

In order to determine the Lagrangian multiplier, it is first necessary to assume that the weight vector A is the smallest, so that most of the sample points lie within the error of the fitted response surface, as shown in Supplementary Figure S2, the sample points are suitable for the slack variables and ζ and $\zeta^{*}$ characterized by W set of regression lines with tolerance ε .

Therefore, the original optimization equation is:

$$L_{\min}=0.5{\|W\|}^{2}+\frac{t}{N}\sum_{i=1}^{N}(\xi_{i}+\xi_{i})$$ (11)

In the formula, t is a constant greater than 0. In order to describe the error correctly, a loss function needs to be defined within the range of (−ε,ε) , which can be expressed as:

$$\begin{array}{l} L_{\varepsilon }=0\forall |f\left(x\right)-y|<\varepsilon \\ L_{\varepsilon }=|f\left(x\right)-y|-\varepsilon \end{array}$$ (12)

Equation (11) can be rewritten as:

$$L=0.5{\|W\|}^{2}+t\sum_{i=1}^{N}\left(\bar{l}\left(\xi_{i}\right)+\bar{l}\left(\xi_{i}^{*}\right)\right)$$ (13)

#### 4.3.2 Response Surface Fitting

**FIGURE 5:**
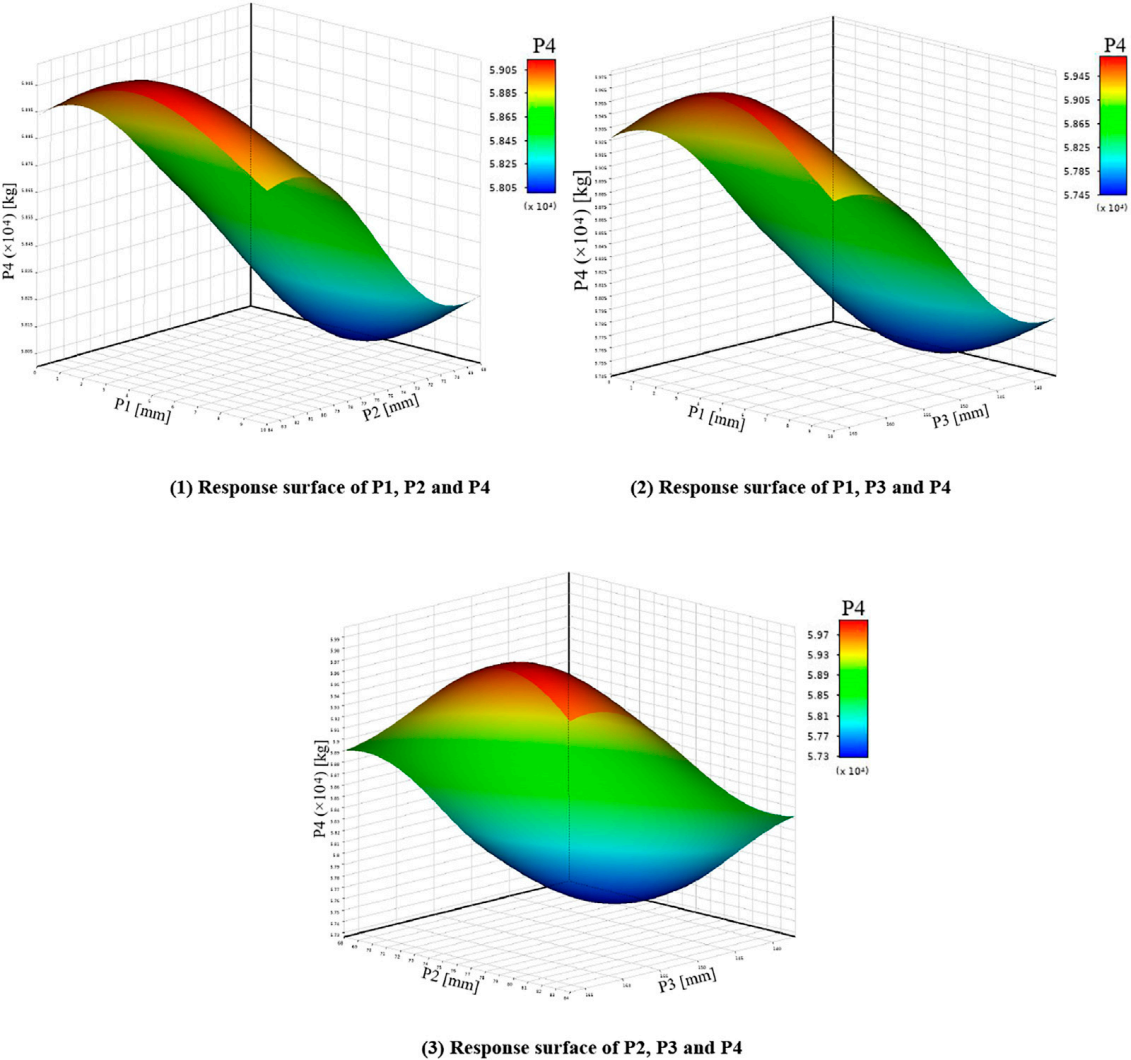
Response surfaces of P1, P2, P3 and P4.

**FIGURE 6:**
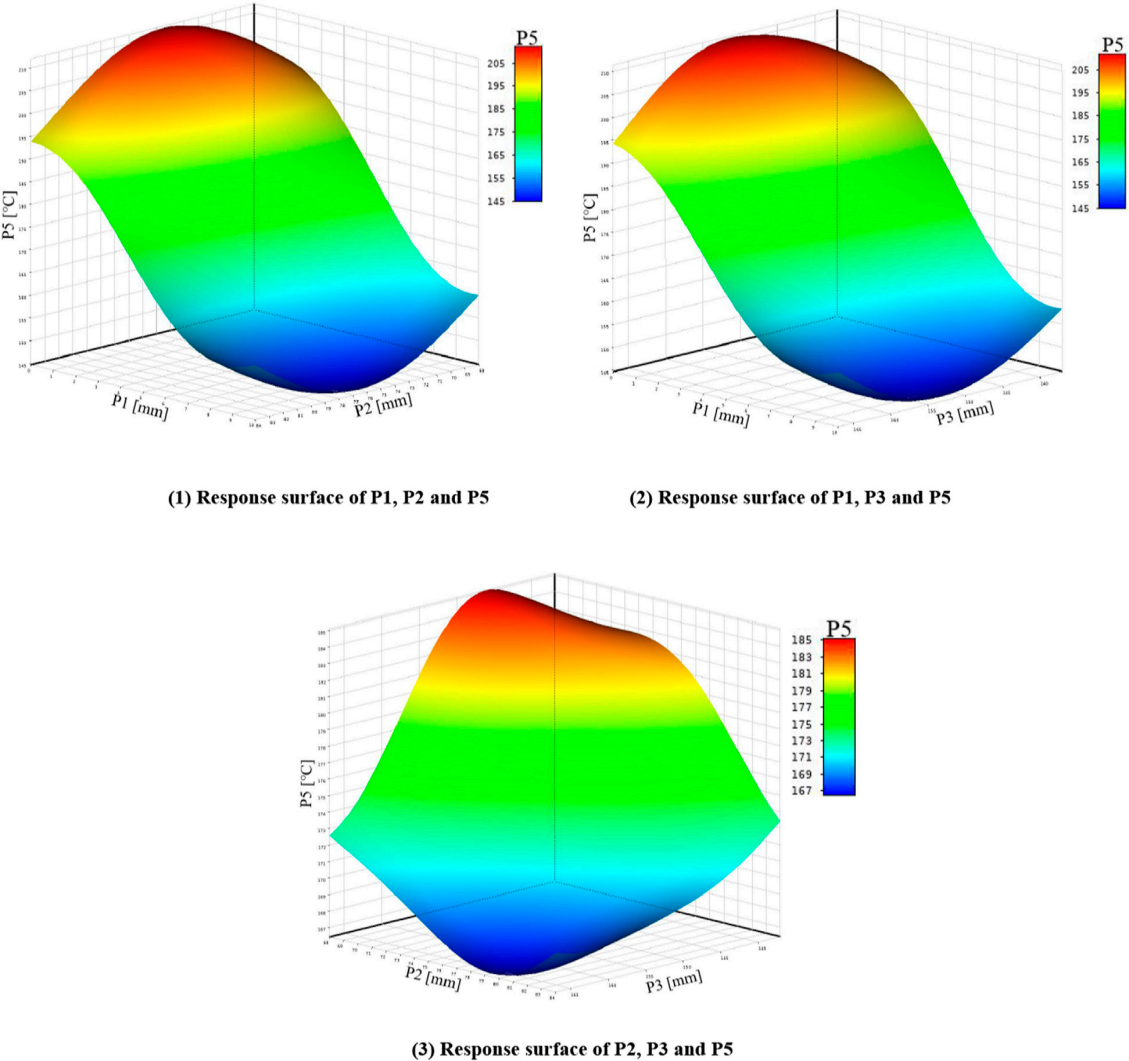
Response surfaces of P1, P2, P3 and P5.

**FIGURE 7:**
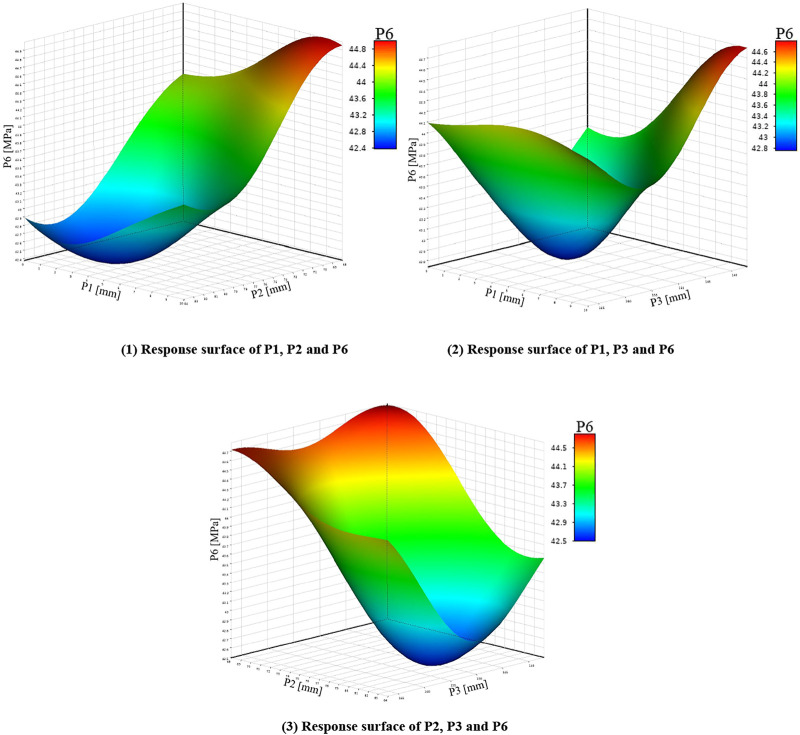
Response surfaces of P1, P2, P3 and P6.

Before fitting the response surface, it is necessary to establish sample points. The commonly used sample points are to adopt certain rules to generate sample points within a certain range. In this paper, the parameters calculated in Table 3 are taken as the sample points and brought into the finite element model. The fitting method adopts the non-parametric regression method with good support for high nonlinearity, and generates the relationship between the input parameters and the output parameters. As shown in Figures 5–7 below. In order to improve the recognition degree of the chart, this paper sets the corresponding numbers of design variables and optimization objectives, as shown in Supplementary Table S2.

**FIGURE 8:**
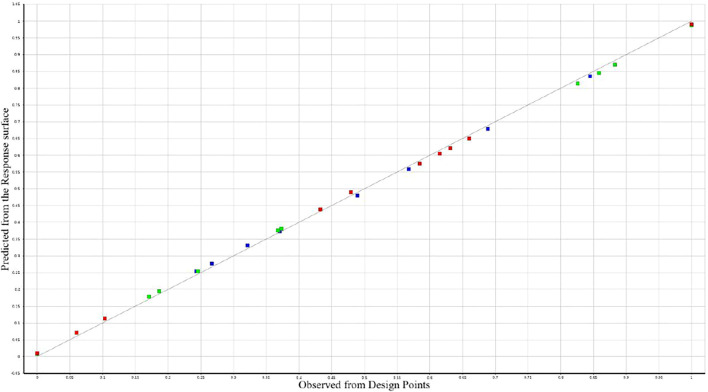
Goodness of fit second-order response surface.

Figure 8 reflects how well the designed output variables fit in the response surface model. The red squares represent the ladle mass, the green squares represent the maximum temperature of the ladle shell and the blue squares represent the maximum stress in the lining. The predicted value of the response surface of each objective function is basically consistent with the observed value of the experimental design point, and they are gathered near the identification line. It shows that the establishment of the response surface model is reasonable and meets the actual design requirements.

## 5 Multi-Objective Genetic Algorithm Optimization and Verification

### 5.1 Overview of Genetic Algorithms

Biological evolution is a very magical process. It produces excellent species through the laws of nature such as selection, elimination, and mutation (Jankauskas and Farid, 2019; Majumder and kar, 2019). Genetic algorithm (GA) is a computer intelligence algorithm abstracted from the selection and evolutionary process of nature (Netjinda et al., 2015; Ngo et al., 2016).

GA forms a random search algorithm with the characteristics of “generate + test” by fully simulating natural selection and genetic mechanisms (Ram et al., 2019; Shastri et al., 2019). The genetic algorithm first determines the fitness function according to the solution space, then determines the initial population and obtains the fitness value of the individual, and judges the obtained results. When the fitness value reaches the optimal solution in the solution space, the algorithm ends. When the optimal solution conditions are not met, enter the next stage to generate a new population through genetic operations, then return to the previous stage to obtain the fitness value of the individuals in the new population, and judge the obtained results, and so on until the requirements are met. (Tanweer and suresh, 2016; Sharifi and Mojallali, 2019; Talbi, 2019).

The basic genetic algorithm consists of basic processes such as coding, fitness function, and genetic operators (selection, crossover, and compilation). Coding is to use a certain coding mechanism to convert objects into strings of specific symbols in a certain order. Just as the study of biological inheritance starts with chromosomes, the genetic algorithm determines the quality of an individua (Huang and He, 2020;Huang and fu, 2021; Duan and Sun, 2021). The basic flow of the genetic algorithm is shown in Supplementary Figure S3 shown.

The general steps of a genetic algorithm are as follows:

1) Use random or other heuristic methods to generate an initial population pop(1),t:=1 with N chromosomes;
2) For each chromosome $pop_{i}\left(t\right)$ in population pop(t) , calculate its fitness value $f_{i}=fitness\left(pop_{i}\left(t\right)\right)$ ;
3) If the stopping condition is met, the algorithm stops; otherwise, some chromosomes are randomly selected to form a new population newpop(t+1) with a set probability or other methods;
4) Crossover with a certain probability to generate some new chromosomes and obtain a new population crosspop(t+1) ;
5) Change a gene of the chromosome with a small mutation probability to form mutpop(t+1):t:=t+1 , and become a new population pop(t)=mutpop(t+1) , and return to step 2.

### 5.2 Mathematical Model of Multi-Objective Optimization Problem

The multi-objective optimization (Mop) problem is to seek a set of design variables $x_{1},x_{2},\cdots ,x_{n}$ that satisfy the constraints and a vector function composed of sub-objective functions, so that the decision maker can accept all sub-objective values. The objective function is a specific description of the system performance of the design variables, and its mathematical model can be expressed as formula (14):

$$\begin{array}{l} Minf\left(x\right)={\left[f_{1}\left(x\right),f_{2}\left(x\right),\cdots ,f_{n}\left(x\right)\right]}^{T},x\in X\\ g_{i}\left(x\right)\geq 0,i=1,2,\cdots ,p\\ h_{j}\left(x\right)=0,j=1,2,\cdots ,q \end{array}$$ (14)

In general, formula (14) is called a mathematical model of multi-objective optimization problems, in which $X=\left(x_{1},x_{2},\cdot \cdot \cdot ,x_{n}\right)$ , n variables $x_{1},x_{2},\cdot \cdot \cdot ,x_{n}$ are decision variables, and the vector x composed of decision variables is called decision vector; n (>1 and an integer) numerical value The objective function $f_{i}\left(x\right)=f_{i}\left(x_{1},x_{2},\cdot \cdot \cdot ,x_{n}\right)$ is the objective function, and the vector formed by the objective function is called the vector objective function; $g_{i}\left(x\right)$ and $h_{j}\left(x\right)\;$ are called the constraint function.

### 5.3 Basic Concepts of Multi-Objective Optimization

The optimal solution of multi-objective optimization problem is generally called Pareto optimal solution. In formula (14), if solution $x^{1}\in X$ is superior to all other solutions, then $x^{1}$ is said to be the optimal solution of the multi-objective optimization model. If there is a solution $x^{1}\in X$ that does not make all $f_{n}\left(x\right)$ optimal, but there is no better solution than $x^{1}$ , then $x^{1}$ is called a non-inferior solution of the multi-objective optimization model, also known as the Pareto optimal solution (Huang and chen, 2022).

Definition 14) Given a multi-objective optimization problem f(X) , its optimal solution is defined as

$$f\left(X^{*}\right)=\underset{X\in \Omega }{opt}f\left(X\right)$$ (15)

$$\mathrm{in},f:\Omega \to R^{r}$$ (16)

where Ω is the feasible solution domain that satisfies the constraints in formula (14).

Definition 15) For a multi-objective problem min f(X) , define $X^{*}\in \Omega$ to be the optimal solution (Pareto optimal solution), if for any X∈Ω , the following conditions are satisfied:

$$\underset{i\in I}{\wedge }\left(f_{i}\left(X\right)=f_{i}\left(X^{*}\right)\right)$$ (17)

Or there is at least one j∈I,I=(1,2,⋅⋅⋅,r) such that

$$f_{j}\left(X\right)>f_{j}\left(X^{*}\right)$$ (18)

Definition 16) For a multi-objective problem min f(X) , if $X^{*}\in \Omega$ , and there is no other $X^{\#}\in \Omega$ , such that

$$f_{j}\left(X^{*}\right)\geq f_{i}\left(X^{\#}\right)$$ (19)

holds, and at least one of them is a strict inequality, then $X^{*}$ is said to be the Pareto optimal solution of min f(X)

Definition 17) For a multi-objective problem min f(X) , for any $X_{1},X_{2}\in \Omega$ :

If $f\left(X_{1}\right)\leq f\left(X_{2}\right)$ , then $X_{1}$ is said to be superior to $X_{2}$ ;

If $f\left(X_{1}\right)<f\left(X_{2}\right)$ , then $X_{1}$ is said to be superior to $X_{2}$ .

From the definition (17), it can be known that there is often more than one solution that satisfies the Pareto optimal solution condition, but a Pareto optimal solution set.

Definition 18) For a multi-objective problem min f(X) , its optimal solution set is defined as:

$$P^{*}=\left\{X^{*}\right\}=\left\{X\in \Omega ,\exists X^{\prime }\in \Omega ,f_{j}\left(X^{\prime }\right)\leq f_{j}\left(X\right),\left(j=1,2,...,r\right)\right\}$$ (20)

The optimization process of MOGA is to find the current optimal individual (optimal solution) in each generation of evolutionary population, which is called the non-dominated solution in the generation, or the non-dominated solution. Inferior solution (non-inferior solution); the current set of all optimal individuals is called the non-dominated set (NDS) of this generation, and the core of the algorithm is to make the non-dominated set continuously approach the theoretical Pareto of the objective function. The optimal solution set, and finally reaches the optimal.

### 5.4 Selection of Multi-Objective Optimization Algorithm

In multi-objective optimization problems, the solution of a multi-objective optimization problem is often a non-inferior solution. Because the objectives are contradictory and mutually constrained, and the improvement of one objective is often at the cost of the loss of other objectives, and it is impossible to make each objective reach the optimal solution (Luo and Sun, 2020). Multi-objective optimization algorithms are divided into traditional optimization algorithms and intelligent optimization algorithms, among which traditional optimization algorithms can be further divided into weighting, constraint and linear regression methods, and intelligent optimization algorithms are evolutionary algorithms (EA) and particle swarm algorithms (PSO). Evolutionary algorithms include multi-objective genetic algorithms (MOGA), non-dominated ranking genetic algorithms (NSGA) and NSGA-ΙΙ, as shown in Supplementary Figure S4. Traditional optimization algorithms generally obtain one of the Pareo solution set at a time, while using intelligent algorithms to solve, more Pareto solutions can be obtained, and these solutions constitute an optimal solution set called Pareto optimal solution (Liu L. et al., 2022).

In this paper, MOGA is used to calculate the structure optimization problem of the new ladle refractory lining, and the Design Exploration module in ANSYS Workbench provides a convenient way to optimize the design, and is widely used in practical engineering analysis. According to the needs of experimental conditions, various parameters to be analyzed and designed are included in the analysis process, which is beneficial to design such as optimization analysis. These include related parameter system, response surface system, objective-driven optimization system, Six Sigma analysis system, etc. The optimization algorithm of the ladle lining adopts the multi-objective genetic algorithm in the response surface optimization method. The multi-objective optimization flowchart is shown in Supplementary Figure S5.

### 5.5 Establishment of Multi-Objective Optimization Model

Take the working layer thickness, permanent layer thickness, and insulating layer thickness of the ladle lining as input parameters. On the basis of the response surface method, using MOGA to optimize the ladle lining structure. Set the number of objective functions to 3, the decision variable to 3, set the maximum and minimum values of the decision variable, the population size to 100, the number of iterations to 20, the crossover probability to 90%, the mutation probability to 1/3. The objective function of this optimization is the fitness function. The maximum equivalent force on the refractory lining is within the permissible stress range as a constraint. The mathematical model for ladle lining optimization is shown in Eq. 21:

$$\left\{ \begin{array}{l} Min\left(\alpha ,\beta ,\chi \right)=f\left(H_{1},H_{2},H_{3}\right)\\ 0\leq H_{1}\leq 10\\ 68\leq H_{2}\leq 84\\ 137\leq H_{3}\leq 167\\ \beta \leq 48MPa \end{array}\right.$$ (21)

In the formula: $H_{1}$ is the thickness of the working layer; $H_{2}$ is the thickness of the permanent layer; $H_{3}$ is the thickness of the insulating layer; α is the maximum temperature of the ladle shell; β is the maximum equivalent stress of the ladle lining; χ is the total mass of the ladle.

### 5.6 Optimization Results and Verification Analysis

#### 5.6.1 Results of the Optimization

The multi-objective optimization problem can only be solved by coordinating the trade-offs of each objective so that each sub-objective can be optimized. For the refractory lining layer size optimization problem, the maximum ladle shell temperature and the maximum refractory lining stress are reduced and the ladle mass is reduced under the premise that the maximum stress in the refractory lining is within the permissible stress range. The optimal size optimization is to find the best balance between the two opposing problems of ladle insulation and longevity. The Pareto solution candidates obtained by the multi-objective genetic algorithm are shown in Table 4.

**TABLE 4:** Pareto solution candidate points.

Candidate Points	Insulation Layer Thickness (mm)	Permanent Layer Thickness (mm)	Working Layer Thickness (mm)	Ladle Quality (kg)	Maximum Temperature of Ladle Shell (°C)	Maximum Stress of lining(MPa)
1	7.87	77.36	151.19	58,639	148.06	43.44
2	8.02	76.20	148.61	58,225	149.25	43.66
3	6.55	79.05	145.38	58,224	160.74	43.56

It can be seen from Table 4 that the maximum stress of the refractory lining of the three groups of candidate points is within the allowable stress range. The magnitude of the refractory lining stresses did not differ significantly among the three groups of candidate points. Candidate point 1 and candidate point 2 have the best insulation performance, but the mass of the ladle at candidate point 2 is better than that of the ladle at candidate point 1. Therefore, candidate point 2 is selected as the optimal design point.

The optimized calculation results of the ladle refractory lining structure are shown in Table 5.

**TABLE 5:** Optimization results of lining structure.

	Variable	Before Optimization	Optimized	
Design variable	Insulation layer thickness	0	8.02	
	Permanent layer thickness	81	76.20	−5.93%
	Working layer thickness	152	148.61	−2.23%
Objective function	Maximum temperature of ladle shell (°C)	214.46	149.25	−30.41%
	Maximum equivalent stress of ladle lining (MPa)	43.94	43.66	−0.64%
	Ladle total mass (kg)	59,020	58,225	−1.35%

Among them, the thickness of the insulating layer changed the most, from 0 mm before optimization to 8.02 mm. The thickness of the permanent layer is reduced from 81 mm before optimization to 76.20 mm, a change of 5.93%. The thickness of the working layer has the smallest change, which is reduced from 152 mm before optimization to 148.61 mm, and the change range is 2.23%. The maximum temperature of the ladle shell is reduced from 214.46°C before optimization to 149.25°C, and the change range is the largest, reaching 30.41%. The maximum equivalent stress of the ladle lining is reduced from 43.94 MPa before optimization to 43.66 MPa, with a small change of 0.64%. The total mass of the ladle is reduced from 59020 kg before optimization to 58225 kg, with a change range of 1.35%, which achieved the purpose of optimization.

#### 5.6.2 Validation Analysis

In order to verify the reliability of the above optimization results, this section uses numerical simulation methods for thermal stress analysis and comparison with the insulation performance of the unoptimized ladle refractory lining. The same analysis steps, interactions, and boundary conditions as in Section 3 are set in turn.

**FIGURE 9:**
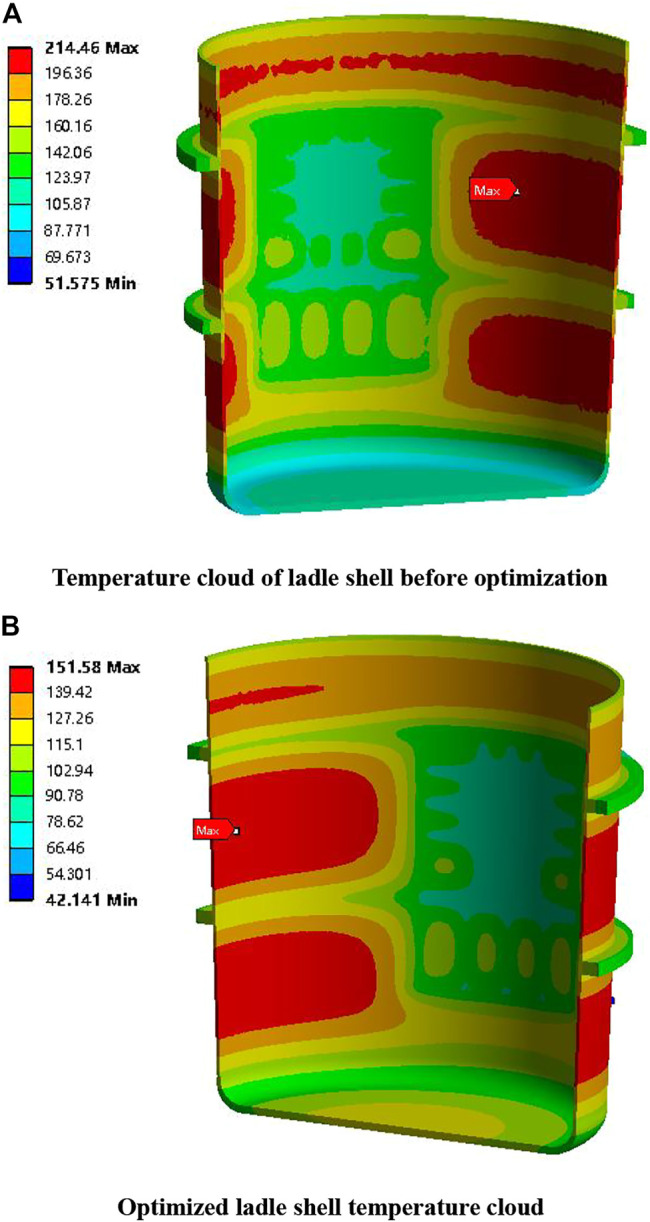
Comparison of stress distribution in ladle lining before and after optimization.

**FIGURE 10:**
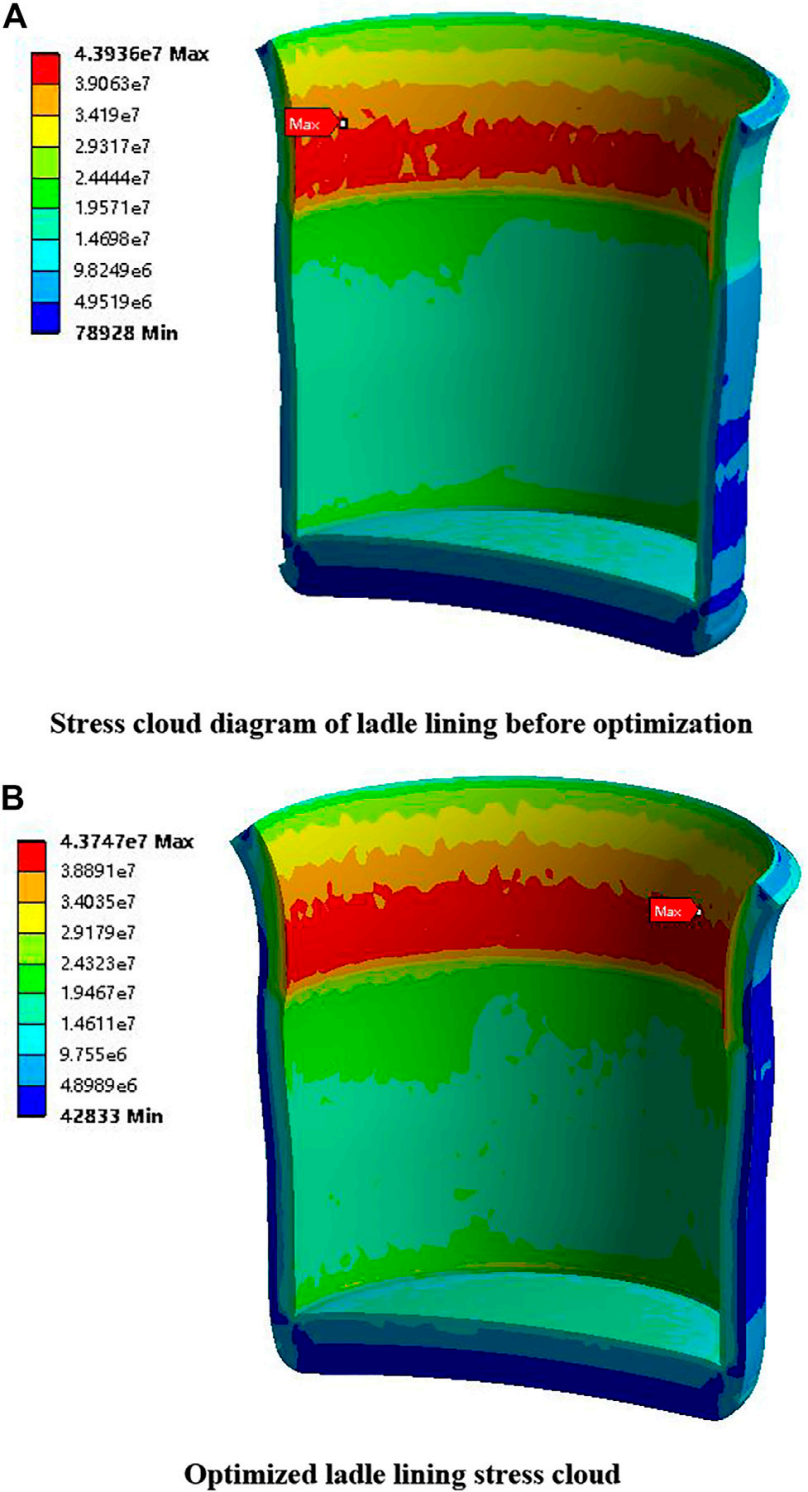
Comparison of ladle shell temperature distribution before and after optimization.

Figure 9 shows the comparison of the temperature distribution of the ladle shell before and after optimization. The maximum temperature of the ladle shell after optimization is 151.58°C. The maximum temperature of ladle shell obtained after optimization by multi-objective genetic algorithm is 149.25°C, which is a good fit with the results of finite element software analysis, and the error is only 1.53%, both of which are lower than the maximum temperature of 214.46°C of ladle shell before optimization. Figure 10 shows the comparison of stress distribution in the ladle lining before and after optimization. The maximum equivalent stress of the ladle liner after optimization is 43.75 MPa, and the maximum equivalent stress of the ladle liner after optimization by multi-objective genetic algorithm is 43.66 MPa, which is a good fit with the results of the finite element software analysis, with an error of only 0.21%, and is lower than the maximum equivalent stress of the ladle liner before optimization, which is 43.94 MPa. The optimization model is verified to be correct and the effect of optimization is achieved.

## 6 Conclusion

The optimal design of ladle refractory lining based on finite element analysis software is an organic combination of mechanical design and computer simulation technology. In this paper, ANSYS Workbench software is used to parametrically model the ladle as a solid, and the model is imported using the seamless connection between the software. Then the optimal filled space design method (OSFD) is used to complete the design of the test program. Then the parametric design platform established by the secondary development technology in Workbench is used to establish models with different parameter combinations, and thermal stress analysis is performed for each group of models to complete the acquisition of sample points. Finally, the response surface model is established by using nonparametric regression, and the Pareto optimal solution is found by genetic algorithm, and the following results are obtained.

1) The optimized insulation layer thickness is increased from 0 to 8.02 mm, the permanent layer thickness is reduced from 81 to 76.20 mm, and the working layer thickness is reduced from 152 to 148.61 mm.
2) The maximum ladle shell temperature is reduced from 214.46 to 149.25°C, the maximum equivalent force of the lining is reduced from 43.94 to 43.66 MPa, and the total ladle mass is reduced from 59,020 kg to 58,225 kg after optimization.
3) The structure of the ladle lining is optimized by using multi-objective genetic algorithm in response surface optimization method to improve the service life and insulation performance of the ladle.

For the multi-objective problem, although each sub-objective cannot reach the optimum at the same time, the use of MOGA to obtain the Pareto Frontier can provide the ideal design parameters based on the actual trade-off weight relationship of each objective, which improves the design efficiency. This optimization method of finding the optimal solution avoids repeated iterative calculations of the whole finite element model, saves computation time while ensuring the solution accuracy, and provides a reference method for improving the insulation performance and lightweighting performance of the ladle.

## Data Availability

The original contributions presented in the study are included in the article/supplementary material, further inquiries can be directed to the corresponding authors.
