# Supplementary material for: Multi-Objective Optimization Design of Ladle Refractory Lining Based on Genetic Algorithm
Source: Front Bioeng Biotechnol. 2022 Jun 15;10:900655. doi: 10.3389/fbioe.2022.900655 (PMC9240744; doi:10.3389/fbioe.2022.900655)
Supplement: Supplementary file 1 [file DataSheet1.docx]

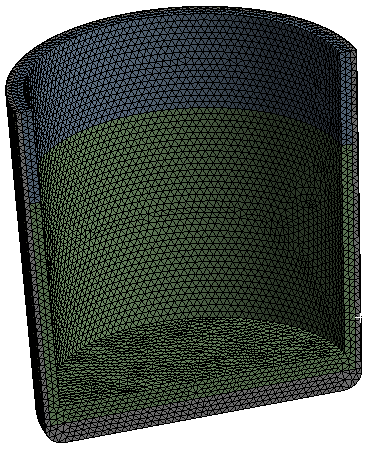


**Figure S1**


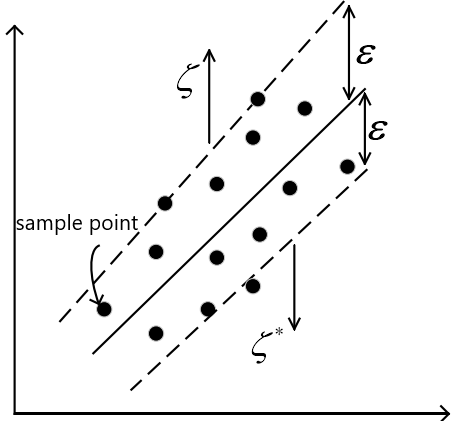


**Figure S2**

**Figure S3**

Multi-objective optimization algorithm

Traditional Optimization Algorithms: Weighted, Constrained, and Linear Regression

Intelligent optimization algorithm

Evolutionary Algorithms（EA）

particle swarm algorithm（PSO）

multi-objective genetic algorithm（MOGA）

Non-dominated sorting genetic algorithm（NSGA）

NSGA-Ⅱ

**Figure S4**

**Figure S5**

**Table S1**

| Optimization variables | Initial value/mm | Variation range/mm |
| --- | --- | --- |
| Insulation layer thickness H_1_ | 0 | 0~10 |
| Permanent layer thickness H_2_ | 81 | 68~84 |
| Working layer thickness H_3_ | 152 | 137~167 |

**Table S2**

| Variable | Insulation layer thickness H_1_ | Permanent layer thickness H_2_ | Working layer thickness H_3_ | Ladle quality | Maximum temperature of steel cladding | Maximum stress of refractory lining |
| --- | --- | --- | --- | --- | --- | --- |
| Serial number | P1 | P2 | P3 | P4 | P5 | P6 |
